# Mechanotransduction-Epigenetic Coupling in Pulmonary Regeneration: Multifunctional Bioscaffolds as Emerging Tools

**DOI:** 10.3390/ph18101487

**Published:** 2025-10-02

**Authors:** Jing Wang, Anmin Xu

**Affiliations:** 1 Department of Physiology, School of Medicine, Southeast University, Nanjing 210009, China; 2 Jiangsu Provincial Key Laboratory of Critical Care Medicine, Zhongda Hospital, School of Medicine, Southeast University, Nanjing 210009, China; 3 Laboratory of Environmental Medicine Engineering, Ministry of Education, School of Public Health, Southeast University, Nanjing 210009, China

**Keywords:** multifunctional scaffolds, mechanobiology, epigenetic regulation, pulmonary fibrosis, CRISPR/dCas9s, lung regeneration, biomaterials, mechanotransduction, tissue engineering

## Abstract

Pulmonary fibrosis (PF) is a progressive and fatal lung disease characterized by irreversible alveolar destruction and pathological extracellular matrix (ECM) deposition. Currently approved agents (pirfenidone and nintedanib) slow functional decline but do not reverse established fibrosis or restore functional alveoli. Multifunctional bioscaffolds present a promising therapeutic strategy through targeted modulation of critical cellular processes, including proliferation, migration, and differentiation. This review synthesizes recent advances in scaffold-based interventions for PF, with a focus on their dual mechano-epigenetic regulatory functions. We delineate how scaffold properties (elastic modulus, stiffness gradients, dynamic mechanical cues) direct cell fate decisions via mechanotransduction pathways, exemplified by focal adhesion–cytoskeleton coupling. Critically, we highlight how pathological mechanical inputs establish and perpetuate self-reinforcing epigenetic barriers to regeneration through aberrant chromatin states. Furthermore, we examine scaffolds as platforms for precision epigenetic drug delivery, particularly controlled release of inhibitors targeting DNA methyltransferases (DNMTi) and histone deacetylases (HDACi) to disrupt this mechano-reinforced barrier. Evidence from PF murine models and ex vivo lung slice cultures demonstrate scaffold-mediated remodeling of the fibrotic niche, with key studies reporting substantial reductions in collagen deposition and significant increases in alveolar epithelial cell markers following intervention. These quantitative outcomes highlight enhanced alveolar epithelial plasticity and upregulating antifibrotic gene networks. Emerging integration of stimuli-responsive biomaterials, CRISPR/dCas9-based epigenetic editors, and AI-driven design to enhance scaffold functionality is discussed. Collectively, multifunctional bioscaffolds hold significant potential for clinical translation by uniquely co-targeting mechanotransduction and epigenetic reprogramming. Future work will need to resolve persistent challenges, including the erasure of pathological mechanical memory and precise spatiotemporal control of epigenetic modifiers in vivo, to unlock their full therapeutic potential.

## 1. Introduction

Pulmonary fibrosis (PF) is a chronic progressive lung disease that leads to respiratory failure. Its pathogenesis involves irreversible alveolar destruction, impaired capillary barrier function, pathological extracellular matrix (ECM) accumulation, and progressive tissue stiffening [1,2]. Current therapies are palliative, alleviating symptoms without halting disease progression or reversing fibrosis. Approved drugs including pirfenidone and nintedanib only slow functional decline but fail to reverse established fibrosis or regenerate functional alveoli [3,4,5]. This therapeutic limitation arises from a self-sustaining fibrotic microenvironment, where biochemical abnormalities (e.g., persistent profibrotic cytokines) converge with aberrant biomechanics such as elevated matrix stiffness [6,7]. Innovative approaches capable of co-targeting these dual drivers are therefore urgently needed. Therefore, clarifying how these biochemical and biomechanical imbalances act on key reparative cells is essential for designing therapies that can reverse fibrosis.

Given the limitations of current therapies, a deeper understanding of the cellular and molecular mechanisms driving PF progression is crucial. Alveolar type II epithelial Cells (AT2 Cells) serve as the major progenitor population responsible for lung repair and regeneration. Following lung injury, AT2 Cells proliferate and differentiate into AT1 Cells to restore gas-exchange surfaces [8,9]. However, chronic exposure to profibrotic mediators disrupts this process. Transforming growth factor-β1 (TGF-β1) plays a central role by driving fibroblast-to-myofibroblast transdifferentiation via Smad2/3 phosphorylation and by promoting epithelial–mesenchymal transition (EMT) [10,11]. In addition, TGF-β1 engages secondary pathways such as Wnt/β-catenin and Notch, which further amplify fibrogenesis and reinforce pathological ECM deposition [12,13]. These signaling perturbations converge with epigenetic dysregulation, forming a multifaceted barrier to AT2 regeneration. In addition to these signaling abnormalities, epigenetic dysregulation has emerged as a pivotal driver of persistent AT2 dysfunction. DNA hypermethylation and histone deacetylation maintain fibroblast activation and compromise AT2 cell plasticity [14,15]. The reversible nature of these epigenetic modifications enables viable therapeutic targeting. Emerging evidence indicates that mechanical signals are transmitted from focal adhesions through actomyosin and the linker of nucleoskeleton and cytoskeleton (LINC) complex to the nucleus, where they reshape chromatin and thereby alter epigenetic states that affect AT2 plasticity. Moreover, the effects of epigenetic dysregulation are closely intertwined with mechanical abnormalities in the fibrotic niche. Critically, increased tissue stiffness can trigger and stabilize pathological epigenetic alterations via mechanotransduction signaling in fibrotic microenvironments. This mechanical information is then converted into stable epigenetic marks that lock cells into a profibrotic state. This process establishes a mechanically self-reinforcing barrier specific to fibrotic disease. This barrier perpetuates fibrosis progression by maintaining fibroblasts in an activated state and restricting AT2 regenerative potential [16,17,18].

To effectively disrupt this self-reinforcing barrier, therapeutic strategies must simultaneously target both its mechanical and epigenetic components. Multifunctional bioscaffolds represent promising vehicles to reverse pathological remodeling of the fibrotic niche [19,20]. The mechanical properties of these scaffolds can be precisely engineered to replicate physiological stiffness gradients. Soft regions that mimic healthy alveoli generally inhibit YAP/TAZ activity, whereas stiff regions promote RhoA/ROCK signaling; the underlying mechanisms are detailed in Section 2.2 and Section 2.3 [21,22]. Moreover, these constructs enable spatiotemporal delivery of regenerative factors (such as keratinocyte growth factor) and antifibrotic compounds, thereby coordinating tissue repair with fibrosis suppression [23,24,25]. Scaffolds also function as precision carriers for epigenetic modulators. Biodegradable polymers delivering DNA methyltransferase (DNMT) inhibitors (designated DNMTi) reverse hypermethylation of antifibrotic genes including BMP7. Histone deacetylases (HDACs) are enzymes that regulate epigenetic modifications, while their inhibitors, termed histone deacetylase inhibitors (HDACi), exert therapeutic effects by blocking HDAC activity. Hydrogel-encapsulated HDACi restore histone acetylation to block myofibroblast persistence [26,27,28,29]. These integrated scaffold systems convert passive structural templates into bioactive matrices that actively direct tissue regeneration [30,31]. Crucially, by simultaneously providing physiological mechanical cues and delivering epigenetic modulators, scaffolds offer a unique strategy to co-target and disrupt the intertwined mechano-epigenetic drivers of fibrosis. Preclinical studies using bleomycin (BLM)-induced models, precision-cut lung slices and organoids demonstrate scaffold efficacy in reversing established fibrotic remodeling [32,33,34].

This review presents an integrated mechano-epigenetic framework for scaffold-based lung regeneration. We analyze scaffold-guided mechanical control of cell fate based on current evidence. Systematic examination of epigenetic reprogramming via targeted chromatin modifier delivery is then presented. Particular emphasis is placed on the demonstrated synergy between scaffold mechanics and epigenetic drug release. Finally, clinical translation pathways for stimuli-responsive systems are evaluated. This framework enables novel therapeutic strategies to disrupt the self-sustaining fibrotic cascade in PF.

## 2. Mechanotransduction via Scaffold Mechanics

**Figure 1:**
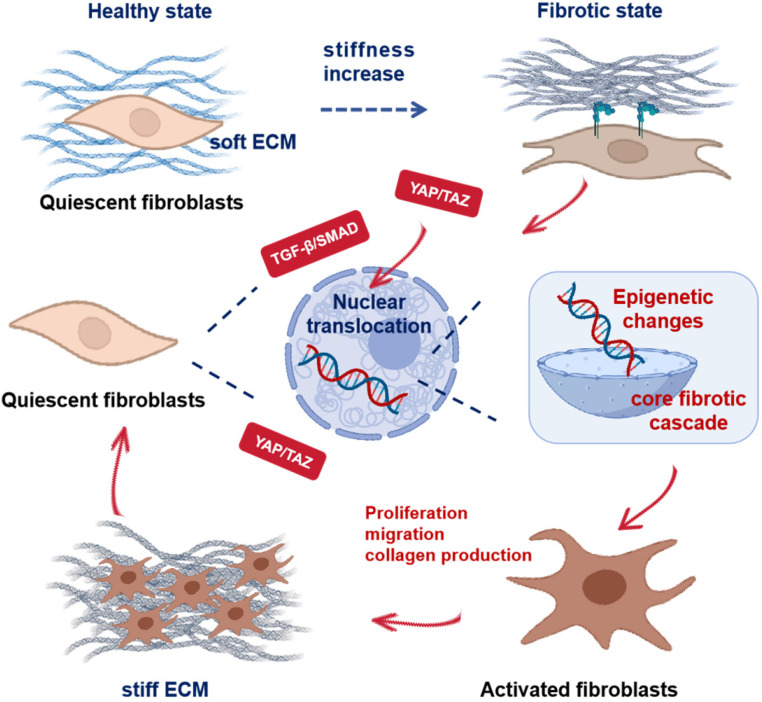
The schematic of the transition from a healthy, compliant matrix to persistent fibrotic remodelling in the lung driven by mechanical cues. Quiescent Cells reside within a soft extracellular matrix (ECM). Progressive collagen deposition and crosslinking elevate tissue stiffness. Cells sense mechanical changes via integrin-mediated adhesions. This triggers the activation of mechanosensitive regulators such as YAP and TAZ, as well as profibrotic TGF-β and SMAD signalling. These factors translocate to the nucleus and drive transcriptional reprogramming. Accompanying epigenetic modifications such as DNA methylation and histone alterations stabilize profibrotic gene expression and promote fibroblast and myofibroblast activation. This leads to increased proliferation, migration and collagen production. Activated Cells further remodel and contract the ECM, increasing stiffness and establishing a self-sustaining positive feedback loop that perpetuates fibrosis.

This section explores how scaffold-mediated mechanical cues regulate cellular responses essential for lung regeneration (Figure 1). We first establish fundamental principles of scaffold mechanical design including stiffness gradients and dynamic strain simulation. Subsequent analysis explores mechanotransduction mechanisms from focal adhesion assembly to cytoskeletal tension transmission. Finally, we analyze how integrated signaling pathways coordinate cell fate decisions and matrix remodeling. Together, these mechanobiological strategies establish targeted guidance of tissue morphogenesis to restore functional lung architecture in PF.

### 2.1. Scaffold Elastic Modulus and Stiffness Gradient Design

In lung tissue engineering, scaffold mechanical properties critically determine cellular behavior. Physiological alveolar ECM stiffness ranges from 1 to 5 kPa [35,36], whereas fibrotic extracellular matrix exhibits pathological stiffening beyond 20 kPa [37,38]. Biocompatible hydrogels accurately replicate this stiffness spectrum through controlled crosslinking density modulation. These materials allow researchers to mimic both healthy and early fibrotic microenvironments, providing optimal conditions for epithelial cell function and progenitor cell maintenance [39,40].

To simulate advanced fibrotic pathology, polymeric networks and nanocomposite reinforcements enable scaffold stiffening exceeding 20 kPa [41,42]. This approach recapitulates disease progression. It shows how excessive stiffness drives profibrotic cellular programs. Understanding these mechanisms is directly relevant for designing interventions to halt or reverse fibrosis. Such stiffened microenvironments authentically recapitulate disease progression by activating fibroblast-to-myofibroblast transdifferentiation. This process enhances alpha-smooth muscle actin expression and increases contractile activity [43,44]. Stiff substrates additionally upregulate fibrosis-associated genes including *collagen type I alpha 1 chain (Col1a1)* and *Actin alpha 2 (ACTA2)* while simultaneously impairing endogenous ECM remodeling capacity [44,45]. Pathological stiffness further disrupts basement membrane integrity, altering epithelial cell polarity and inhibiting regenerative repair mechanisms [46,47]. These effects on fibroblast activation and epithelial regeneration are partly mediated by mechanotransduction pathways such as YAP/TAZ and RhoA/ROCK, as described in Section 2.2 and Section 2.3.

Spatially controlled stiffness gradients generated via 3D bioprinting or microfluidic technologies establish continuous transitions from soft to stiff regions [48,49]. These engineered gradients mimic the heterogeneous stiffness found in fibrotic lungs. They enable region-specific investigation of epithelial and fibroblast responses, which is clinically relevant. Within these platforms, scaffold regions of specific stiffness elicit distinct cellular responses. Compliant zones at 1–5 kPa modeled on healthy alveoli promote epithelial cell adhesion and proliferation [50,51,52]. This regenerative effect is partly mediated via YAP/TAZ signaling, as elaborated in Section 2.2. Conversely, rigid domains exceeding 20 kPa that emulate fibrotic foci drive profibrotic cellular differentiation. These effects involve mechanotransduction pathways, including RhoA/ROCK, discussed in Section 2.3.

### 2.2. Dynamic Stretch and Aberrant YAP Activation

Physiological stretch supports alveolar epithelial homeostasis, whereas pathological stretch drives aberrant YAP activation linked to fibrosis. Beyond static stiffness matching, recapitulating physiological breathing-induced dynamic stretch is essential for alveolar epithelial barrier integrity and surfactant secretion [53,54]. In vitro models apply cyclic tensile strain to elastomeric scaffolds that replicate physiological breathing patterns observed in vivo [55,56]. Dynamic stimulation enhances integrin-mediated cell–matrix coupling and promotes continuous reorganization of intracellular stress fibers, leading to increased actin turnover rates [57,58]. Such remodeling optimizes tension transmission through actin-myosin networks, thereby facilitating activation of mechanotransduction effectors and supporting alveolar morphogenesis [59,60]. This biomechanical priming promotes efficient activation of downstream mechanotransduction effectors (such as YAP/TAZ), facilitating alveolar morphogenesis. Physiological stretch maintains alveolar epithelial homeostasis. Pathological stretch triggers aberrant YAP activation, leading to maladaptive repair and fibrosis. These findings inform scaffold design. Biomaterials that replicate normal cyclic breathing support epithelial function. They also limit pathological YAP/TAZ activity and improve the therapeutic efficacy of scaffolds in fibrotic lungs.

In fibrotic lungs, excessive stretch disrupts homeostasis and induces pathological nuclear YAP accumulation [61,62]. Activation of the IL-6–SFK–YAP signaling axis compromises epithelial fluid balance and barrier function, ultimately promoting fibrotic remodeling [63]. Idiopathic pulmonary fibrosis (IPF) patients exhibit persistent pathological YAP activation in airway epithelial Cells. This dysregulated YAP signaling directly contributes to epithelial barrier disruption [64,65]. Sustained YAP/TAZ hyperactivity across diverse cell types further impairs regeneration and accelerates fibrosis progression [22,66,67].

### 2.3. Focal Adhesions and Cytoskeletal Tension Transmission

Cells transduce external mechanical cues into biochemical responses through focal adhesions and the cytoskeletal network. When scaffold stiffness exceeds physiological ranges, enhanced phosphorylation occurs in core focal adhesion components including focal adhesion kinase (FAK) and adaptor protein Paxillin [68,69,70]. FAK phosphorylation drives α-SMA expression and myofibroblast differentiation, orchestrating TGF-β–mediated cytoskeletal reorganization and fibrogenic gene programming [71,72,73,74]. Pharmacological blockade of FAK inhibits these fibrogenic responses, highlighting its essential regulatory role in fibrosis.

Parallel activation of the RhoA/ROCK signaling cascade stimulates extensive stress fiber polymerization, significantly amplifying cytoskeletal contractile forces [75,76]. Force-stabilized networks strengthen cell-scaffold integration via integrin microaggregates while propagating mechanical signals to nuclei through actomyosin contractility and LINC complex-mediated nuclear reshaping [27,77,78,79]. This mechanical signaling axis modulates chromatin architecture, establishing a force-sensitive epigenetic regulation mechanism [80,81].

### 2.4. Integrated Mechanosignaling in Fibrosis

In PF, the mechanical microenvironment undergoes pathological changes. Elevated matrix stiffness and aberrant cellular tension characterize this dysregulated state. These altered mechanical forces activate key mechanosensitive signaling pathways, thereby driving fibrogenesis [38,82]. Elevated matrix stiffness (>20 kPa) induces fibroblast autocrine TGF-β1 secretion. TGF-β1 signaling through SMAD2/3 phosphorylation then drives fibroblast-to-myofibroblast transdifferentiation and excessive collagen deposition, constituting the core fibrotic cascade [45,83]. Elevated matrix stiffness and cytoskeletal tension activate YAP/TAZ, driving their nuclear translocation. This triggers TEAD-dependent transcription of target genes such as PCNA, CTGF, and CYR61, which together enhance fibroblast proliferation, migration, survival, and myofibroblast differentiation [84,85,86]. Increased stiffness also potentiates Wnt/β-catenin signaling. Stabilized β-catenin translocates to the nucleus and upregulates profibrotic genes, including CCND1 (Cyclin D1) and MYC (c-Myc) [87,88]. Collectively, activation of these mechanosensitive pathways expands and activates fibroblasts within the stiffened microenvironment, driving ECM deposition and fibrogenesis. Pathological ECM stiffening converges on the TGF-β/SMAD and YAP/TAZ pathways, creating a self-reinforcing signaling network that drives progressive lung remodeling and functional decline [89,90,91].

Beyond mechanotransduction, mechanical stress has been shown to remodel epigenetic states. This induces persistent mechanical memory through modified histone states and concurrent chromatin architectural reorganization [92,93,94]. Such mechano-driven epigenetic reprogramming plays a critical role in maintaining fibrogenic cell phenotypes. Pathological tissue stiffness is associated with upregulation of epigenetic enzymes such as DNA methyltransferase 1 (DNMT1) and G9a (EHMT2), promoting promoter DNA hypermethylation and repressive histone modifications. Consequently, antifibrotic gene expression is suppressed, accelerating fibrogenesis. These epigenetic alterations induce chromatin compaction and transcriptional silencing, thereby maintaining fibroblast profibrotic activation [95,96]. In liver fibrosis models, combined G9a (EHMT2) and DNMT1 inhibition reverses these epigenetic aberrations [95,97]. This intervention reactivates protective gene expression and potently suppresses fibrogenesis, indicating strong therapeutic translatability. Mechanical stimuli orchestrate histone methylation states via cytoskeletal force transmission to the nucleus [98,99,100]. This mechano-sensitive epigenetic reprogramming strongly influences cellular phenotypic commitment and functional specialization. Collectively, these findings demonstrate the close interplay between mechanical forces and epigenetic regulation in fibrosis pathogenesis. This mechanistic hierarchy provides a foundation for scaffold-mediated epigenetic reprogramming strategies aimed at disrupting pathological mechanical memory, detailed in Section 3.

## 3. Epigenetic Drug Delivery via Scaffolds

Mechanical cues regulate cellular behavior through canonical signaling pathways while modifying epigenetic programs in lung fibroblasts and epithelial Cells. This process reinforces profibrotic gene expression [46,101]. Accumulating evidence indicates that matrix stiffness and cyclic strain remodel DNA methylation profiles and histone modification patterns, driving persistent myofibroblast differentiation [99,102]. This creates a mechanically reinforced epigenetic barrier that sustains fibrosis. To circumvent this mechanically stabilized epigenetic barrier, scaffold-based delivery systems can be designed for localized administration of small-molecule inhibitors or nucleic acid therapeutics within fibrotic niches. The following section details strategies to deliver DNMT and HDACi via hydrogel and nanofiber scaffolds, alongside emerging approaches targeting non-coding RNA and methyl-binding proteins, for spatiotemporal control of epigenetic reprogramming in PF. These strategies aim to directly reverse the mechano-induced epigenetic dysregulation described in Section 2.4.

### 3.1. DNMT Inhibitor Carrier Design

Pathological hypermethylation of anti-fibrotic gene promoters, driven by aberrant DNMT upregulation, constitutes a well-established therapeutic target in PF [103,104]. Critically, this hypermethylation is amplified by pathological mechanical cues. To address this mechanism, localized delivery systems show significant promise, specifically gelatin methacryloyl (GelMA) hydrogels for the DNMTi 5-azacytidine (5-AZA). This approach utilizes GelMA’s enzyme-responsive biodegradation to achieve sustained drug release within fibrotic niches exhibiting elevated matrix metalloproteinase expression. The controlled release kinetics align with the prolonged exposure requirements for effective epigenetic modulation [105,106,107]. Supporting evidence from varied delivery vehicles demonstrates 5-AZA’s ability to suppress DNMT function and restore physiological methylation patterns [103,108]. Moreover, GelMA’s biomimetic properties such as RGD motifs facilitate cellular interactions that may synergize with epigenetic reprogramming [109,110,111].

GelMA hydrogels provide critical advantages as localized epigenetic therapy carriers. Their tunable physicochemical characteristics allow tailored control of drug release kinetics required for sustained epigenetic reprogramming [112,113]. The hydrophilic hydrogel matrix of GelMA enables effective encapsulation of hydrophilic therapeutics such as 5-AZA, concurrently preventing degradation of susceptible compounds during transit. This protective capability overcomes inherent stability limitations of nucleoside analogs in systemic circulation [114,115,116]. GelMA’s biomimetic ECM structure facilitates cell–matrix interactions extending beyond RGD-mediated adhesion. These interactions could potentiate cellular sensitivity to epigenetic agents through mechanotransduction pathway regulation, a process actively sustaining fibrotic progression [42,117]. Experimental validation confirms that microenvironmental reprogramming enhances therapeutic outcomes of epigenome-targeting agents in pathological tissues. Collectively, these attributes establish GelMA-mediated 5-AZA delivery as a viable strategy for targeted DNMT inhibition in fibrotic lung tissue. This approach directly counters stiffness-induced hypermethylation, resolving the core epigenetic barrier underlying fibrosis.

### 3.2. HDACi Carrier Design

The inherent biocompatibility and tunable degradation of electrospun polycaprolactone (PCL)/collagen nanofibers position them as viable substrates for sustained drug release [118,119]. Combining PCL’s mechanical robustness with collagen’s bioactivity, these scaffolds maintain drug release for weeks. Such sustained delivery supports the efficacy of diverse regenerative therapeutics, including antibiotics and growth factors [120,121]. For instance, PCL-based airway stents incorporating antitumor agents have shown clinical potential in reducing tissue hyperplasia through prolonged local drug exposure [122,123]. Collagen-enhanced nanofibrous scaffolds potentiate wound healing by confining therapeutic agents at disease loci with precise spatiotemporal control [124,125]. The HDACi trichostatin A (TSA) and suberoylanilide hydroxamic acid (SAHA) attenuate fibrotic remodeling and oncogenesis via selective epigenetic remodeling [126,127,128]. Importantly, they counteract the histone deacetylation driven, in part, by pathological mechanical stress. Despite their therapeutic potential, the clinical translation of HDAC inhibitors is hampered by challenges including short systemic circulation and dose-limiting toxicities. To address these limitations, nanoencapsulation strategies have been developed to improve HDACi pharmacokinetic profiles. Evidence suggests that polymer-based nano-delivery systems, in particular, offer advantages in enhancing drug stability and promoting tissue-specific accumulation.

The strategy of integrating HDACi within PCL/collagen scaffolds builds upon established foundations in drug delivery and antifibrotic therapy. Direct reports on this specific combination for fibrosis treatment remain limited. However, technical feasibility is supported by analogous drug-carrier systems. Electrospun PCL matrices have successfully encapsulated structurally similar hydrophobic compounds, demonstrating compatibility with the physicochemical properties of typical HDACi [129,130]. Collagen components provide cell-adhesive motifs including integrin-binding domains, which may enhance drug accumulation and cellular uptake of HDACi at pathological sites. This effect has been observed in tumor microenvironment studies where collagen-functionalized carriers promote drug internalization by stromal or tumor Cells [131,132]. Furthermore, the nanofibrous scaffold architecture delivers physical cues known to directly regulate fibroblast spreading behavior and phenotypic transition [133,134]. This modulation establishes a foundation for creating a microenvironment favorable for targeted epigenetic therapy in fibrotic lesions.

Emerging evidence suggests such combinatorial approaches could address critical limitations in chronic disease management. Localized HDACi delivery via biodegradable scaffolds may circumvent systemic toxicity while enabling sustained modulation of fibrotic cascades, a strategy aligned with current trends in precision nanomedicine. Further optimization of release kinetics through material engineering could enhance temporal control over epigenetic reprogramming events, positioning this platform as a promising frontier for antifibrotic intervention. This approach directly targets the mechano-induced histone hypoacetylation contributing to the fibrotic epigenetic barrier.

### 3.3. Novel Epigenetic Targets for Scaffold-Based Intervention

Novel epigenetic regulators beyond classical DNMTi and HDACi provide opportunities for precision antifibrotic therapy through engineered scaffold-based delivery systems [135,136]. For instance, let-7 miRNA delivery suppresses the BTB and CNC homology 1 (BACH1)-enhancer of zeste homolog 2 (EZH2)-MYC signaling axis in murine fibrotic lungs, inhibiting alveolar epithelial cell reprogramming toward pro-fibrotic phenotypes [137,138]. Genetic lineage tracing demonstrates that let-7 restoration suppresses EZH2 expression in AT2 Cells, with concomitant reduction in global H3K27me3 histone methylation in fibrotic lungs [137]. Chromatin immunoprecipitation analyses further reveal that this epigenetic remodeling reactivates transcriptional programs essential for cell differentiation, inhibiting transition toward pro-fibrotic intermediates [139,140]. Although these associations are established, the precise causal relationship between let-7 mediated EZH2 downregulation and site-specific H3K27me3 reduction requires further investigation. Scaffold systems enabling controlled nucleic acid release could deliver let-7 mimics to exploit this epigenetic cascade for coordinated editing in fibrotic lungs.

Regarding another target, pathological MeCP2 overexpression induces epigenetic silencing of WIF1 via promoter hypermethylation, driving fibroblast-to-myofibroblast transdifferentiation in human fibrotic tissues [141,142]. Nanofibrous scaffolds achieve sustained, localized siRNA delivery for long-term gene silencing and effective fibrotic response modulation [143,144]. However, experimental evidence remains unreported for MeCP2-targeted siRNA nanofiber scaffolds specifically acting through WIF1 demethylation and Wnt/β-catenin pathway suppression in high-impact studies. These findings validate combined MeCP2-targeted siRNA and advanced scaffold systems for precise epigenetic intervention in fibrotic diseases. Essential future work must resolve the molecular mechanisms governing this targeted strategy.

The therapeutic efficacy of these approaches depends on scaffold design parameters. Optimized nanofiber architectures provide controlled release kinetics for nucleic acid therapeutics, and surface modifications enhance cellular internalization efficiency at fibrotic lesions [145,146]. Notably, biomaterial physical properties directly influence epigenetic effector activity, since substrate stiffness modulates histone modification enzymes in mesenchymal Cells [80,147,148]. Dual-targeting scaffolds co-delivering two nucleic acid therapeutics such as miRNAs or siRNAs achieve synergistic modulation of fibrotic pathways. These systems demonstrate enhanced cellular reprogramming and gene silencing effects relative to single-agent delivery in preclinical models. Cardiac fibroblast reprogramming exemplifies this advantage with dual miRNA-loaded scaffolds outperforming single miRNA systems [149,150,151]. Collectively, scaffold-mediated epigenetic targeting of regulators like let-7 and MeCP2 demonstrates a precision antifibrotic strategy. Critical knowledge gaps persist regarding mechanical memory erasure efficiency and in vivo spatiotemporal control of epigenetic modifiers (such as DNMTs and siRNAs). Resolving these challenges through intelligent biomaterial design will accelerate clinical translation of mechano-epigenetic therapies.

### 3.4. Dual-Drug Synergy Strategy

Combined treatment with 5-AZA and TSA synergistically regulates epigenetic pathways including DNA methylation and histone acetylation, reducing fibrotic progression. Preclinical kidney fibrosis models demonstrate that 5-AZA/TSA co-treatment reactivates key antifibrotic genes and signaling pathways, reducing fibrotic progression [152,153]. This dual epigenetic modulation enhances antifibrotic efficacy by concurrently targeting DNA methylation and histone acetylation. While current scaffold systems lack mechanical gradient-enabled sequential release of 5-AZA/TSA, integration of spatiomechanical cues with epigenetic drug actions represents a strategic advancement for antifibrotic biomaterial design.

**Figure 2:**
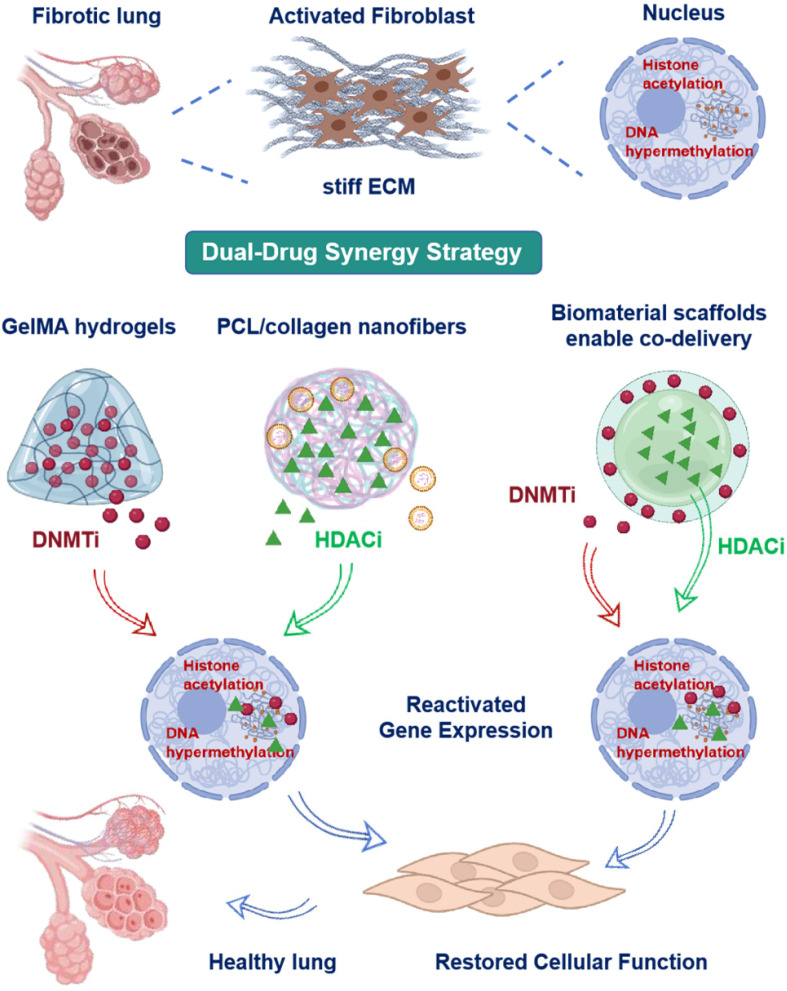
Dual Drug Synergy Strategy for Epigenetic Reprogramming in Pulmonary Fibrosis (PF). The top panel depicts fibrotic lung tissue containing activated fibroblasts and aberrant epigenetic alterations, such as disrupted histone acetylation and DNA hypermethylation. The central panel presents biomaterial-based delivery systems, including gelatin methacryloyl (GelMA) hydrogels for the delivery of DNMT inhibitors (DNMTi), polycaprolactone (PCL)/collagen nanofibers for HDAC inhibitors (HDACi), as well as composite scaffolds engineered for co-delivery. The bottom panel demonstrates how combined treatment with DNMTi and HDACi reverses disease-associated epigenetic modifications, reactivates silenced antifibrotic genes, and facilitates both functional and structural restoration of lung tissue toward a physiological state. The figure highlights the promise of biomaterial-mediated co-delivery systems for synergistic epigenetic therapy in PF.

Current evidence indicates synergistic epigenetic regulation from combined DNMTi and HDACi treatment may enhance CpG island demethylation and histone acetylation effects [154,155,156]. Biomaterial scaffolds enable co-delivery of epigenetic agents, potentially enhancing synergistic efficacy through spatiotemporal control [30,157,158]. Supporting this concept, fibrotic lung models demonstrate substantially reduced collagen I deposition, reestablished E-cadherin expression and restored alveolar architecture. However, studies report limited direct evidence comparing these systems to single-drug approaches. Recent studies establish that temporally and spatially controlled release systems dynamically modulate cell fate and gene expression. Mechanical gradient integration further enhances this regulatory capacity [81,159,160,161]. These advances suggest promise for overcoming mechano-epigenetic barriers, though synergistic disruption mechanisms require further validation. This approach interrupts fibrotic feedback loops and remodels alveolar microenvironments via coordinated mechano-epigenetic reprogramming (Figure 2).

## 4. Selected Pulmonary Fibrosis Models for Scaffold Application

The selection of pathophysiologically relevant models is paramount for evaluating scaffold-based interventions in pulmonary fibrosis. While in vivo systems capture systemic complexity, ex vivo platforms offer unprecedented resolution for mechanistic dissection. This section examines complementary models that address translational challenges from target validation to clinical application. These models enable integrated assessment of scaffold biofunctionality across molecular, cellular and organ-level metrics.

### 4.1. Bleomycin-Induced Murine Model

The BLM-induced murine model remains the gold standard for preclinical evaluation of scaffold-based antifibrotic interventions. This model reliably reproduces core pathophysiological features of PF within a controlled time course. Specifically, it recapitulates alveolar epithelial injury, inflammatory cell infiltration and progressive ECM remodeling [162,163]. Decellularized lung matrix (DLM) scaffolds sourced from healthy or fibrotic lungs maintain native three-dimensional architecture. These scaffolds preserve critical ECM ligand composition and physiological biomechanical cues. This preservation establishes DLM as a reproducible platform for investigating cell–matrix interactions and directing targeted recellularization protocols [164,165,166].

Recellularization of DLM scaffolds with epithelial progenitors or fibroblasts promotes cellular adhesion and migration, providing a microenvironment for lung tissue regeneration. While short-term gas exchange improvements have been documented in animal models, long-term functional recovery including sustained pulmonary compliance and gas-exchange capacity remains challenging. Direct in vivo evidence for DLM scaffolds constraining myofibroblastic differentiation or releasing antifibrotic factors is still limited [31,167,168].

These protective outcomes are attributable to the scaffold’s dual functions: restitution of physiological mechanical microenvironments and localized delivery of biochemical signals that modulate profibrotic pathways. Recent work emphasizes the translational potential of ECM-derived hydrogels for attenuating fibrogenic remodeling in BLM models, while highlighting practical considerations for scaffold preparation sterility and recellularization efficiency [169,170,171].

Practical evaluation requires multimodal endpoints. Histomorphometry and collagen quantification such as hydroxyproline assays are essential for assessing matrix burden [172,173]. Respiratory mechanics measured by flexiVent systems and arterial blood-gas metrics provide functional corollaries [174,175]. Scaffold-mediated benefit correlates with reduced collagen deposition and reversal of cellular activation states. This includes decreased alpha-smooth muscle actin (α-SMA) expression and downregulated TGF-β/SMAD signalling, consistent with mechanobiological mechanisms [169,176,177]. To strengthen causal inference, experimental designs increasingly combine DLM implantation with lineage tracing, targeted mechanotransduction perturbations such as *YAP*/*TAZ* inhibition, and epigenetic readouts. This links restored mechanics to transcriptional reprogramming of resident cells [174,178,179,180].

### 4.2. Ex Vivo Lung Slices and Organoids

Precision-cut lung slices (PCLS) and lung organoids occupy a critical intermediate niche between cell culture and whole-animal studies. PCLS retain native multicellular architecture, alveolar–capillary juxtaposition, and local ECM context. This enables direct scaffold application and high-fidelity monitoring of epithelial, mesenchymal and immune responses [178,181,182].

When combined with high-dimensional readouts such as single-cell RNA sequencing, ex vivo perturbations reveal cell type-specific transcriptional trajectories. These permit rapid assessment of scaffold-induced modulation of profibrotic gene programmes including Col1a1, ACTA2 and MMPs alongside epigenetic regulators [101,181,183]. Recent methodologic advances include standardized PCLS preparation and improved cryopreservation workflows. These have expanded assay throughput and reproducibility for mechanistic dissection [178,181].

Lung organoids derived from pluripotent or adult stem cells complement PCLS by enabling longer-term culture. They allow manipulation of stem/progenitor lineage cues and systematic tuning of scaffold properties such as porosity and stiffness gradients [184,185]. Co-culture with multifunctional scaffolds reproduces key morphogenetic events including lumenogenesis and epithelial differentiation. This facilitates controlled screens to identify stiffness ranges and drug-release kinetics favoring regenerative outcomes [186,187,188].

These platforms are particularly valuable for personalized scaffold optimization when using patient-derived organoids. This links ex vivo response phenotypes to potential precision-medicine strategies. Collectively, PCLS and organoid systems accelerate lead selection, reduce animal usage, and provide mechanistic bridges informing in vivo scaffold deployment [189,190].

## 5. Discussion and Future Perspectives

**Figure 3:**
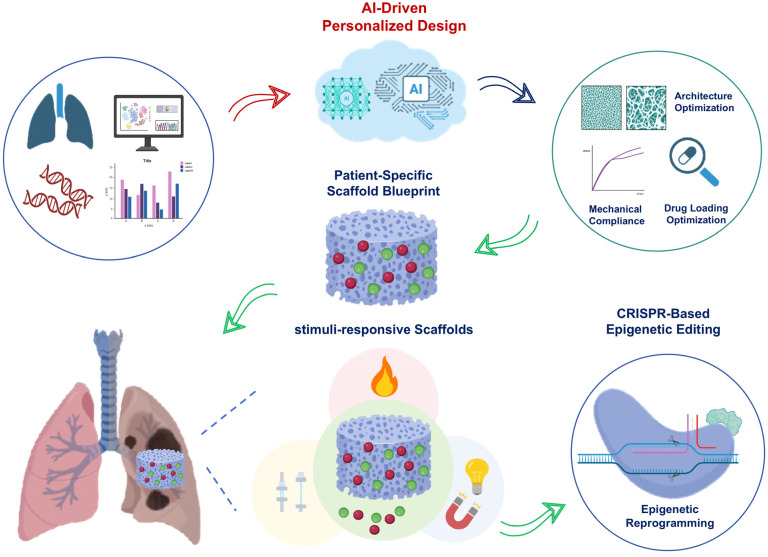
Artificial intelligence (AI)-driven patient-specific scaffold design and epigenetic reprogramming strategy. Clinical and multi-omics data from patients are used to generate tailored scaffold designs, with AI optimizing architectural features, mechanical compliance, and drug loading parameters. The design incorporates stimuli-responsive scaffolds capable of sensing local microenvironmental cues and releasing therapeutics in a feedback-controlled manner. These platforms also facilitate CRISPR-based epigenetic editing alongside pharmacological intervention to achieve targeted reprogramming of disease-associated epigenetic signatures. Subsequent panels illustrate the recovery of cellular functionality and tissue integrity following treatment, highlighting the potential of combining AI-guided biomaterial design, stimuli-responsive delivery, and epigenetic editing for personalized antifibrotic therapy.

The therapeutic potential of multifunctional scaffolds targeting mechanobiology and epigenetics for lung regeneration has been established in preceding sections. While the benefits of these approaches are substantial, several technical challenges remain. Moving forward, translating this potential into clinical practice requires addressing key technological challenges and exploring advanced strategies for scaffold design and functionality. To provide a coherent perspective, this section highlights three interrelated directions: stimuli-responsive scaffolds, CRISPR-based epigenetic editing, and AI-driven personalization. Rather than being considered separately, these strategies are emphasized in terms of their integration (Figure 3).

### 5.1. Stimuli-Responsive Scaffolds

Recent advances have shown that smart scaffolds, constructed from functional polymers and nanocomposites, can sense changes in the pulmonary microenvironment. These changes include factors like oxidative stress and mechanical stretch. Importantly, the scaffolds can dynamically adjust their behavior in response to these sensed changes [191,192,193]. For example, ROS-responsive linkers have been used to build hydrogels. These hydrogels selectively degrade under elevated ROS levels. This degradation enables the on-demand release of antioxidant or antifibrotic drugs specifically within inflamed tissues [194,195,196]. Independent studies demonstrate that ROS-responsive nanoparticles and liposomes can be formulated for inhalation delivery to fibrotic lungs. This strategy exploits the high ROS microenvironment characteristic of these diseased tissues. Consequently, local drug accumulation is improved [197,198,199]. Block copolymers and shape memory polymers have been designed to exhibit reversible deformation under cyclic stretch. This mechanical stimulus mimics the natural breathing cycle. Consequently, these materials can modulate local stiffness in a time-dependent manner [200,201,202]. Recent advances have yielded two-way and light-responsive shape memory polymers (SMPs). These materials offer practical actuation strategies. Such strategies are compatible with physiological strain regimes. Furthermore, they utilize milder activation methods. These features make them attractive for lung applications [203,204]. Triggerable microcapsules embedded in hydrogels can release large protein cargos. These cargos include enzymes or CRISPR effector proteins. Release occurs under specific triggers such as light, magnetic fields, or ROS. This capability demonstrates the feasibility of scaffold-enabled, on-demand epigenetic editing while highlighting the need to carefully optimize delivery systems to ensure efficacy and safety [205,206,207].

Building on these advances in responsive materials, the next section considers how CRISPR-based epigenetic editing provides complementary opportunities for precise regulation at the molecular level.

### 5.2. CRISPR-Based Epigenetic Editing

CRISPR/dCas9-based epigenome editors, such as dCas9–TET1 and dCas9–p300, have matured as powerful tools. These tools can write or erase locus-specific DNA methylation and histone acetylation marks. This capability enables precise control of gene regulation. Critically, it achieves this without altering the underlying genomic sequence [208,209,210]. Systematic epigenome editing studies demonstrate that targeted chromatin modification can produce robust transcriptional responses. Importantly, these responses are context-dependent. This evidence supports therapeutic strategies aimed at the locus-specific reactivation of antifibrotic genes [211,212]. However, efficient and safe delivery of CRISPR effectors to pulmonary tissue remains a significant challenge, limiting translation. Local delivery of CRISPR effectors from biomaterials reduces systemic exposure. It also helps confine editing activity to the intended tissue microenvironment. This targeted approach represents a major translational advantage over systemic vectors [213,214,215]. However, there are significant limitations for CRISPR delivery specifically to lung tissue. These include the dense extracellular matrix and mucus barrier in fibrotic lungs, poor penetration of viral or non-viral carriers, potential immune clearance, and limited retention time at the target site. Overcoming these obstacles requires the development of specialized delivery systems, such as inhalable nanoparticles, hydrogel-based depots, or scaffold-mediated local release, to enhance targeting efficiency and editing efficacy [216,217,218]. Safety considerations for CRISPR-based editing are critical. Key concerns include off-target epigenetic effects, immunogenicity of CRISPR components, and persistent unintended chromatin changes. These risks motivate the adoption of specific mitigation strategies. These strategies include the use of nonviral carriers, transient cargo formats, and inducible activation systems. Collectively, they aim to minimize potential hazards [219,220].

While these delivery and safety challenges remain, artificial intelligence offers a means to optimize scaffold design and integrate CRISPR-based interventions with patient-specific strategies, as discussed in the following section.

### 5.3. Artificial Intelligence-Driven Personalized Design

Artificial intelligence (AI) and machine learning (ML) methods are increasingly applied to key challenges in biomaterial development. These challenges include polymeric biomaterial design, scaffold architecture optimization, and multi-parameter trade-off problems. Significantly, these methods accelerate discovery and design cycles [221,222,223]. Data-efficient ML pipelines integrate key computational and experimental methods. Specifically, they couple finite element modeling, experimental screening, and neural network surrogates. This integration enables constrained multi-objective optimization. The optimization targets scaffold lattices and mechanical properties critical for achieving tissue-specific compliance [224,225]. Reviews and recent applied studies demonstrate the utility of specific machine learning approaches. These approaches include supervised learning, active learning, and Bayesian optimization. They can significantly reduce experimental iterations. Furthermore, they effectively guide patient-specific scaffold customization. In addition, AI-guided design considers key scaffold properties, including mechanical compliance, porosity, and drug-loading capacity, to optimize regenerative performance. Despite these advantages, practical implementation must address reproducibility, scale-up challenges, and integration with regulatory standards. This guidance relies on integration with high-throughput characterization and multi-omics readouts [226,227]. AI further supports the scale-up of scaffold manufacturing. Specifically, it aids in predicting key process parameters for additive manufacturing. Additionally, AI enables defect detection during three-dimensional printing. These capabilities collectively enhance reproducibility and batch quality control. Such improvements are essential for successful clinical translation [228,229]. Collectively, AI-driven approaches bridge computational design with clinical-scale manufacturing, accelerating the translation of mechano-epigenetic scaffolds.

In summary, stimuli-responsive scaffolds, CRISPR-based epigenetic editing, and AI-driven personalized design are best viewed as complementary rather than isolated strategies. Smart scaffolds can serve as delivery platforms for CRISPR effectors, while AI methods can refine scaffold properties and support patient-specific applications. Taken together, the integration of these approaches offers a more effective route toward precision therapies for lung regeneration than any single strategy alone.

## 6. Discussion

PF is a chronic progressive disease initiated by recurrent epithelial injury. This condition is defined by alveolar epithelial cell dysfunction combined with aberrant fibroblast activation, pathological ECM deposition, and sustained inflammatory cytokine release. Progressive destruction of lung architecture and declining compliance characterize advanced PF, ultimately leading to respiratory failure [2,230]. Although current antifibrotic drugs (e.g., pirfenidone, nintedanib) can slow disease progression, they cannot significantly reverse established structural damage [231,232]. Given these limitations, developing novel regenerative strategies that both repair tissue and counteract fibrosis remains a core objective in pulmonary medicine.

Multifunctional bioscaffolds deliver coordinated mechanical, biochemical, and pharmacologic interventions and show promising potential to advance lung regeneration. However, direct mechanistic evidence of scaffold-mediated mechano-epigenetic reprogramming in pulmonary fibrosis remains limited [27,233]. Structurally and physically, scaffold materials can precisely mimic the elasticity and topography of the native alveolar ECM within the physiological stiffness range (1–5 kPa). Modulating both stiffness and nanostructure within this range promotes alveolar epithelial cell proliferation and differentiation, thereby facilitating the regeneration of the gas-exchange interface [165,234]. Mechanical stresses transmitted by the scaffold activate key mechanosensitive pathways, including YAP/TAZ, RhoA/ROCK, and TGF-β/SMAD signaling. This activation regulates critical cellular processes such as cell fate determination and ECM synthesis. Importantly, inhibiting these activated pathways disrupts the pro-fibrotic positive feedback loop, thereby attenuating fibrosis [86,165,235].

As carriers for epigenetic regulation, scaffolds can achieve local, precise release of DNMTi and HDACi. For example, 5-Aza demethylates and reactivates antifibrotic genes such as BMP7 and PPARγ, thereby counteracting stiffness-induced hypermethylation [135,236]. By contrast, HDACi including TSA and SAHA increase H3K9 acetylation, restoring histone marks altered by pathological mechanical cues. This restoration blocks TGF-β1/SMAD signaling and induces myofibroblast apoptosis [237,238]. Coupling these release profiles with mechanical gradients enables spatiotemporal and multi-targeted epigenetic modulation. This combined approach could enhance the therapeutic impact beyond current pharmacological options by reshaping cell fate and transcriptional networks at the tissue level [239].

Three-dimensional scaffolds significantly enhance disease modeling in oncology and cardiovascular research through accurate replication of tissue-specific microenvironments. These platforms actively control cell phenotype specification, transcriptional programming, and cell-ECM signaling dynamics, substantially improving physiological relevance in vitro. Scaffold microarchitecture parameters, specifically fiber diameter, pore geometry, and surface wettability, critically regulate cellular adhesion kinetics, proliferative capacity, migratory patterns, and drug response profiles. Recent studies in glioblastoma and myocardial infarction models demonstrate the necessity for model-specific scaffold customization [240,241,242,243]. Although these findings are encouraging, evidence in pulmonary fibrosis models is still emerging, highlighting the need for further validation.

Despite these advances, direct mechanistic evidence of scaffold-mediated mechano-epigenetic reprogramming in pulmonary fibrosis remains limited. Current high-impact research prioritizes oncological and cardiovascular applications, revealing a critical need to investigate scaffold-driven fibrosis regression pathways. To accelerate clinical translation of scaffold-based therapies, we propose three essential actions. First, establish reproducible manufacturing protocols with robust quality control systems. Second, implement standardized epigenetic assays for quantitative analysis of locus-specific DNA methylation and histone acetylation. Third, complete Good Laboratory Practice compliant toxicology studies and large-animal feasibility assessments before initiating human trials. Addressing these steps will bridge the gap between preclinical promises and practical clinical applications.

Future progress will combine stimuli-responsive scaffolds incorporating photo-responsive polymers, pH-sensitive materials, and hydrolytically degradable systems with CRISPR-based epigenetic editing and single-cell omics-guided target selection. This integration will convert tissue engineering scaffolds from passive structural elements into multifunctional dynamic platforms that actively regulate biological processes. Such systems will disrupt pathological mechano-epigenetic crosstalk through unified sensing and therapeutic intervention modules. Machine learning-driven precision engineering could further optimize these strategies, ensuring scaffold designs are adapted to specific lesions, disease stages, and individual patients.

In summary, multifunctional scaffolds serve as complementary therapeutic tools linking mechanistic discoveries in lung regeneration to clinical implementation. The integrated co-targeting of mechanosignaling and epigenetic reprogramming offers a balanced perspective, combining promise with the recognition of current limitations, and provides a versatile approach for PF and terminal lung diseases. This revised view situates scaffold-based therapies within the broader context of current antifibrotic strategies, highlighting both opportunities and practical considerations for translation.

## 7. Conclusions

Multifunctional scaffolds offer a promising approach for lung regeneration by combining mechanical support with biochemical and epigenetic cues. By simultaneously addressing abnormal mechanotransduction and dysregulated gene expression, these scaffolds may help reverse fibrosis and support tissue repair. The continued development of adaptable, patient-tailored scaffolds could facilitate the translation of these strategies into precision therapies for pulmonary diseases.

## Data Availability

No new data were created or analyzed in this study.
